# Physiological and Structural Changes in Leaves of *Platycrater arguta* Seedlings Exposed to Increasing Light Intensities

**DOI:** 10.3390/plants13091263

**Published:** 2024-04-30

**Authors:** Chunyan Wei, Guangyu Luo, Zexin Jin, Junmin Li, Yueling Li

**Affiliations:** 1 Zhejiang Provincial Key Laboratory of Plant Evolutionary Ecology and Conservation, School of Life Sciences, Taizhou University, Taizhou 318000, China; chunyanw1024@163.com (C.W.); 1000459255@smail.shnu.edu.cn (G.L.); jzx@tzc.edu.cn (Z.J.); lijm@tzc.edu.cn (J.L.); 2 Institute of Ecology, School of Life Sciences, Taizhou University, Taizhou 318000, China

**Keywords:** endangered deciduous shrub, light intensity, phenotypic plasticity, photosynthesis, chlorophyll fluorescence, PNUE, chloroplast ultrastructure

## Abstract

Understanding the light adaptation of plants is critical for conservation. *Platycrater arguta*, an endangered deciduous shrub endemic to East Asia, possesses high ornamental and phylogeographic value. However, the weak environmental adaptability of *P. arguta* species has limited its general growth and conservation. To obtain a deeper understanding of the *P. arguta* growth conditions, we examined the leaf morphology and physiology via anatomical and chloroplast ultrastructural analyses following exposure to different natural light intensities (full light, 40%, and 10%). The findings indicated that *P. arguta* seedings in the 10% light intensity had significantly improved leaf morphological characteristics and specific leaf area compared to those exposed to other intensities. The net photosynthetic rate, chlorophyll (Chl) content, photosynthetic nitrogen use efficiency (PNUE), and photosynthetic phosphorus use efficiency (PPUE) exhibited marked increases at a 10% light intensity compared to both 40% light and full light intensities, whereas the light compensation point and dark respiration levels reached their lowest values under the 10% light condition. With reduced light, leaf thickness, palisade tissue, spongy tissue, and stomatal density significantly decreased, whereas the stomatal length, stomatal width, and stomatal aperture were significantly elevated. When exposed to 10% light intensity, the ultrastructure of chloroplasts was well developed, chloroplasts and starch grain size, the number of grana, and thylakoids all increased significantly, while the number of plastoglobules was significantly reduced. Relative distance phenotypic plasticity index analysis exhibited that *P. arguta* adapts to varying light environments predominantly by adjusting PPUE, Chl b, PNUE, chloroplast area, and the activity of PSII reaction centers. We proposed that *P. arguta* efficiently utilizes low light to reconfigure its energy metabolism by regulating its leaf structure, photosynthetic capacity, nutrient use efficiency, and chloroplast development.

## 1. Introduction

Light is the basis for plant growth, development, and persistence, and represents a primary environmental factor influencing plant distribution and the accumulation of carbon assimilation products [1]. In a weak light environment, plants display the light deficiency phenomenon, which cannot provide sufficient material and energy for the normal growth of plants, thereby inhibiting the growth of seedlings [2,3]. However, strong light stress causes photoinhibition in leaves, induces a large amount of reactive oxygen species accumulation, and leads to the destruction of the structure of the PSII photoreaction center, which is also not conducive to the accumulation of photosynthetic products [4]. Therefore, the appropriate light intensity is essential for the growth of plants. With the increasing attention paid to biodiversity conservation, light adaptation of endangered plants has garnered increasing attention [5,6]. Previous studies found that light is the main factor limiting the seedlings’ establishment of *Tetracentron sinense* [7]. Zhang et al. [6] determined that *Heptacodium miconioides* seedlings were suitable for growth in moderate shade, but their shade tolerance was weak, with their growth being significantly impacted under low light conditions. A study on the endangered plant *Camellia nitidissima* found that it is a shade-adapted plant, and its growth increases alongside reduced light levels [8]. Conversely, prior studies have demonstrated that mild shade can improve the net photosynthetic rate of the endangered plants *Camptotheca acuminata* [9] and *Cercidiphyllum japonicum* [10]. Comprehending the light requirements and adjustments in endangered plants contributes to the protection of natural resources.

To account for varied light conditions, plants have formed a series of response mechanisms that make them adaptive or tolerant to the environment [6,11]. As the main organ of photosynthesis, leaves are very sensitive to changes in the light environment, with a high degree of variability and plasticity. Several studies have demonstrated that plants increase the leaf area and specific leaf areas to improve light utilization efficiency [12], and lower the leaf thickness, palisade tissue thickness, and the ratio of palisade tissue to spongy tissue to increase transpiration [13,14]. Physiologically, some plants elevate their chlorophyll content [15], apparent quantum yield [12], photochemical activity, and primary light energy conversion efficiency of PSII in photosynthesis to increase light energy usage for electron transport [8,13]. Additionally, plants alter the size and density of stomata [6], the number of thylakoids and grana [16], and the morphology of chloroplasts in response to changed light conditions [17]. These changes can improve the ability of carbon acquisition and enhance the adaptability and shade tolerance of plants to unfavorable light environments. Plants adapt to different light intensities changes in a variety of ways. Some studies have shown that shading can affect the pattern of carbohydrate allocation and change the absorption and utilization efficiency of water and nutrients [18,19]. Therefore, the integration of morphological, physiological, and cytological characteristics can provide more comprehensive and accurate information about the light adaptation mechanisms of plants.

*Platycrater arguta* Sieb. et Zucc. (Hydrangeaceae), a monotypic deciduous shrub found in the genus *Platycrater*, is a tertiary relict species [20]. This species has a limited distribution range across 200–600 m altitudes in the valleys or cliffs of eastern China and southern Japan. Studying the continental–island disconnection distribution, phylogeography, phytogeography, and flora is, therefore, critical [21]. *P. arguta* has high horticultural value due to its peculiar flower shape and resistance to pruning [22]. Because of its limited population size, habitat fragmentation, and poor natural regeneration capacity, *P. arguta* has been listed as a second-class protected wild plant in China [23]. Habitat fragmentation has resulted in the growth of *P. arguta* seedlings in highly heterogeneous light conditions. Throughout population regeneration, it is necessary to examine the light intensity suitable for *P. arguta* and its adaption to different light intensities. Recently, studies have focused on the *P. arguta* taxonomic position, molecular phylogeography, community composition, and breeding [21,24,25,26]. However, information related to the impact of light levels on stress responses remains limited.

In this study, we examined the leaf morphology, photosynthetic performance, anatomical structure, and chloroplast ultrastructure of *P. arguta* in response to varied light intensities. Our objectives were (1) to investigate the suitable light intensity for *P. arguta* seedling growth and (2) to explore the possible adaptation mechanism to diverse light intensities in *P. arguta*. The findings provide a theoretical foundation for the conservation and natural re-establishment of *P. arguta*.

## 2. Results

### 2.1. Effects of Light Intensities on Leaf Morphology

**Figure 1:**
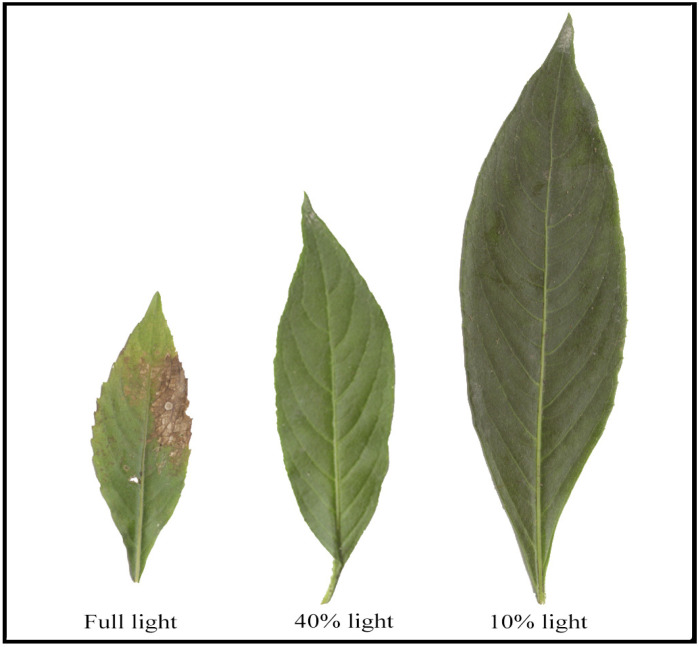
Leaf morphology of *P. arguta* under varied light intensities.

Leaves of *P. arguta* under 10% light intensity were larger and darker than leaves under 40% and full light intensities, while leaves under full light intensity were partially withered (Figure 1). Statistical results showed that the leaves of *P. arguta* under 10% light intensity had significantly higher LL, LW, LA, and SLA than leaves under 40% and full light intensities (Table 1).

### 2.2. Influence of Light Intensities on Photosynthesis Parameters

**Figure 2:**
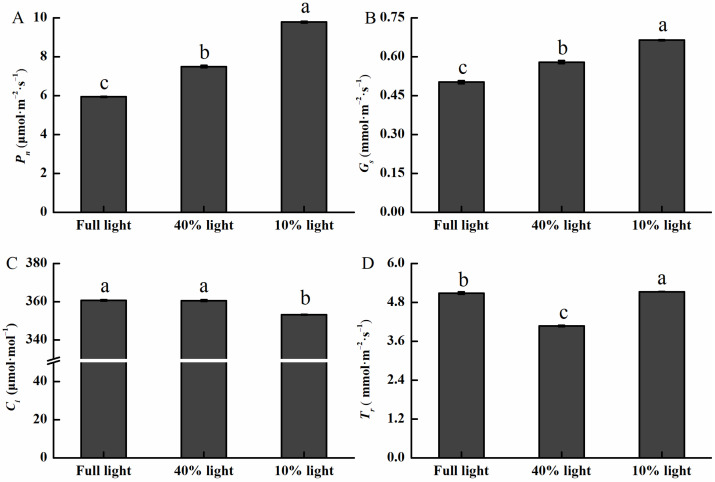
Changes in the net photosynthetic rate (**A**), stomatal conductance (**B**), intercellular CO_2_ concentration (**C**), and transpiration rate (**D**) of *P. arguta* seedlings under varied light intensities. Different lowercase letters indicate significant differences among treatments at the 0.05 level.

Light intensity substantially impacted the photosynthetic parameters of *P. arguta* (Figure 2). The highest value of *P_n_* was observed under 10% light intensity and the lowest under full light intensity (Figure 2A), and the trend of *G_s_* was similar to the trend of *P_n_* (Figure 2B). *C_i_* was the lowest at 10% light condition, contrary to *P_n_* and *G_s_* (Figure 2C). The value of *T_r_* was in the order of 10% light > full light > 40% light (Figure 2D).

**Figure 3:**
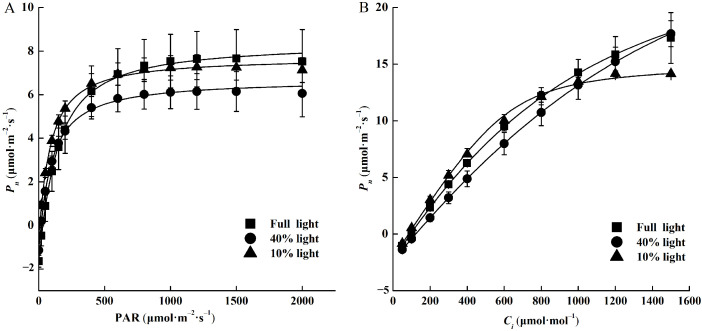
Light response curves (**A**) and CO_2_ response curves (**B**) of *P. arguta* seedlings under varied light intensities.

For light-response curves, when PAR was below 500 μmol·m^−2^·s^−1^, the *P_n_* was higher under 10% light intensity compared to full light and 40% light intensities, whereas, under PAR greater than 500 μmol·m^−2^·s^−1^, the *P_n_* was in the order of full light > 10% light > 40% light (Figure 3A). Further, LCP, LSP, and *R_d_* in 10% light intensity decreased by 80.35%, 47.07%, and 69.09% compared with full light intensity, respectively (Table 2). However, the difference in *P_nmax_* was not significant among different light intensities.

For CO_2_-response curves, in the lower *C_i_* (from 0 mmol·mol^−1^ to approximately 700 mmol·mol^−1^), *P_n_* under varied light intensities was ranked as follows: 10% light > full light > 40% light (Figure 3B). However, alongside elevated *C_i_* (from 700 mmol·mol^−1^ to 1600 mmol·mol^−1^), *P_n_* was lowest under 10% light intensity among three light intensities. Further, *J_max_* and TPU were the lowest under 10% light intensity, and there was no significant difference between full light and 40% light intensities (Table 2). However, the *V_cmax_* did not differ significantly among the three light intensities.

### 2.3. Impacts of Light Intensities on Photosynthetic Pigments and Chl Fluorescence Parameters

**Figure 4:**
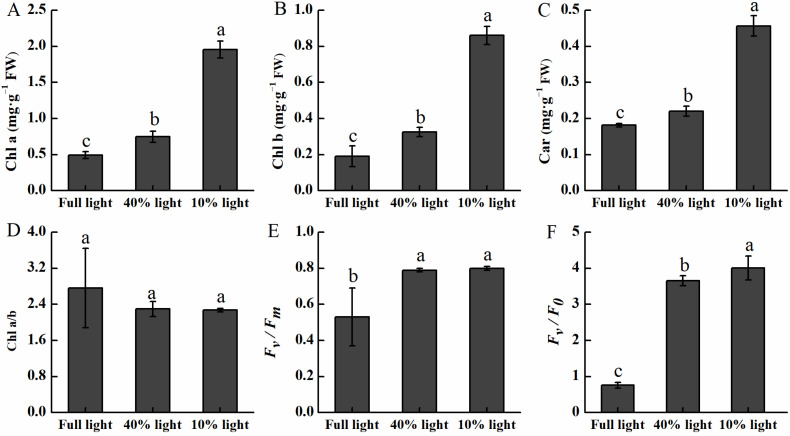
Photosynthetic pigments and chlorophyll fluorescence characteristics of *P. arguta* under varied light intensities. (**A**) Chlorophyll a, (**B**) chlorophyll b, (**C**) carotene, (**D**) chlorophyll a/b, (**E**) maximal photochemical efficiency of PSII, and (**F**) activity of PSII reaction centers. Different lowercase letters indicate significant differences among treatments at the 0.05 level.

Shade increased the photosynthetic pigments and maintained a higher value of the Chl fluorescence parameters of *P. arguta* (Figure 4). Under 40% light and 10% light intensities, Chl a was increased by 51.02% and 297.96%, Chl b was increased by 68.42% and 352. 63%, and Car was increased by 22.22% and 150.00% compared to those under full light intensity, respectively (Figure 4A–C). The highest values of *F_v_*/*F_m_* and *F_v_*/*F_0_* were observed under 10% light intensity. The *F_v_*/*F_m_* and *F_v_*/*F_0_* under 10% light intensity were 50.94% and 427.63% higher than those in full light intensity, respectively (Figure 4E,F).

### 2.4. Impacts of Light Intensities on Nutrient Elements, PNUE, and PPUE

**Figure 5:**
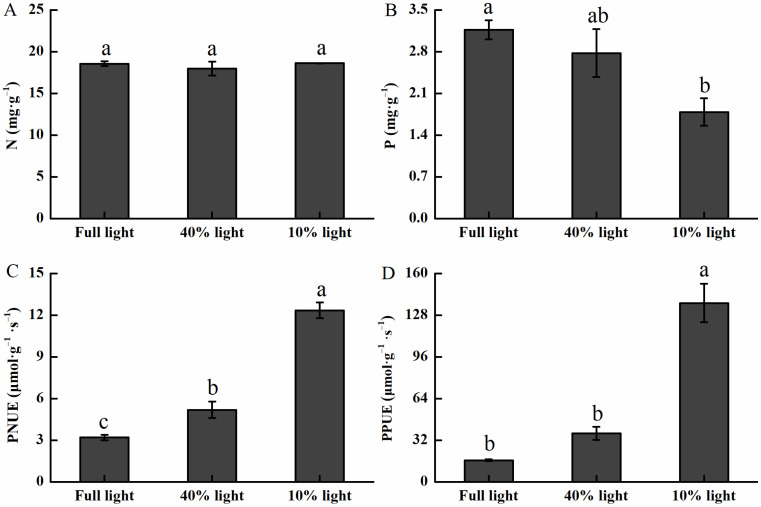
Nutrient elements, PNUE, and PPUE of *P. arguta* leaves under varied light intensities. (**A**) nitrogen, (**B**) phosphorus, (**C**) photosynthetic nitrogen use efficiency, and (**D**) photosynthetic phosphorus use efficiency. Different lowercase letters indicate significant differences among treatments at the 0.05 level.

The contents of PNUE and PPUE increased significantly with the increase in shading, while P content decreased significantly (Figure 5). P content was in the order of full light > 40% light intensity > 10% light intensity (Figure 5B). PNUE and PPUE of *P. arguta* seedlings grown under 10% light intensity were 285.94% and 720.36% larger than those under full light, respectively (Figure 5C,D).

### 2.5. Alterations in Stomatal Properties, Leaf Anatomical Structure, Chloroplast Ultrastructure

**Figure 6:**
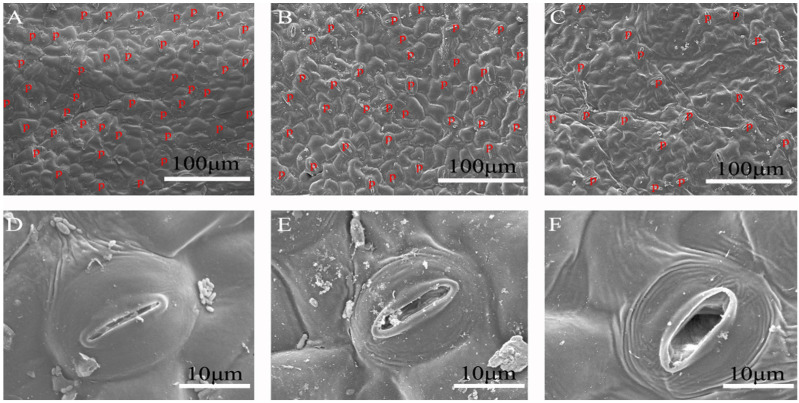
Stomatal structure of *P. arguta* leaves under varied light intensities. (**A**,**D**) Full light, (**B**,**E**) 40% light, and (**C**,**F**) 10% light. P: pores.

Shade reduced the amounts of stomata in *P. arguta* seedlings, but the individual stomata were larger, especially under 10% light intensity (Figure 6). Statistical analyses demonstrated that shade significantly increased SL, SW, and SA but significantly reduced SD (Table 3). Under 10% light intensity, SL, SW, and SA were increased by 9.68%, 9.06%, and 36.40%, respectively, but SD was decreased by 48.78% compared to those under 40% light intensity.

**Figure 7:**
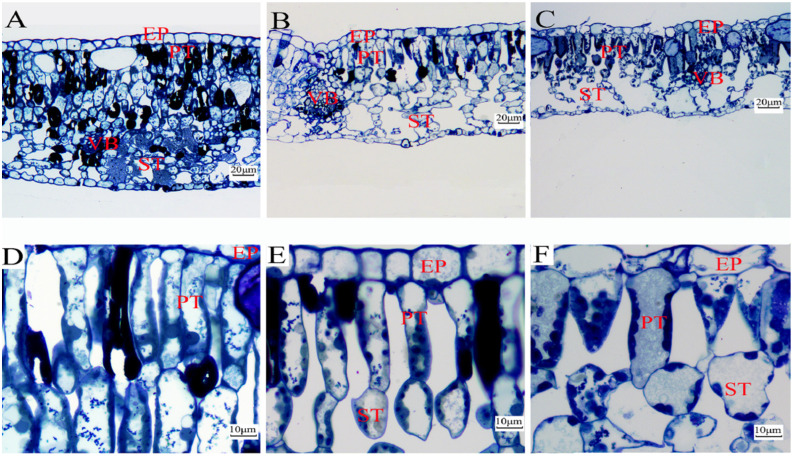
Leaf structural images of *P. arguta* under varied light intensities. (**A**,**D**) Full light, (**B**,**E**) 40% light, and (**C**,**F**) 10% light. EP: epidermis cell tissue; PT: palisade tissue; ST: spongy tissue; VB: vascular bundle.

For leaf anatomical structure, the PT cells were long, narrow, and tightly arranged under full light intensity. In contrast, the PT cells were short, wide, and loosely arranged under 40% light and 10% light intensities (Figure 7A–F). The number of dead or injured cells decreased significantly with increasing shading. Statistical analysis shows that LT, PT, ST, and PT/ST of *P. arguta* seedlings grown under 10% light intensity were 43.53%, 65.07%, 33.35%, and 46.88% smaller than those under full light, respectively (Table 4).

**Figure 8:**
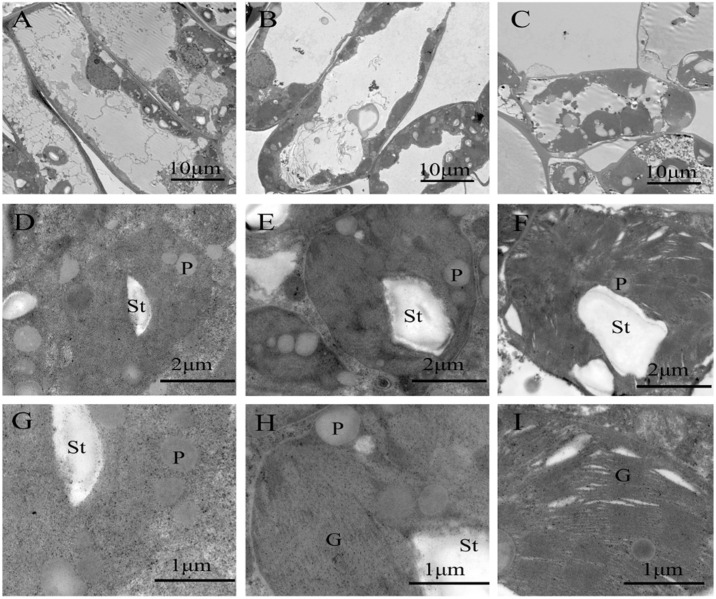
Chloroplast ultrastructure of *P. arguta* leaves under varied light intensities. (**A**,**D**,**G**) Full light, (**B**,**E**,**H**) 40% light, and (**C**,**F**,**I**) 10% light. P: plastoglobules; G: grana; St: starch grain.

The chloroplasts formed in *P. arguta* seedlings were well-developed under shade, especially under 10% light. Under full light intensity, a minority of chloroplasts were distributed in the cells, and a large number of plastoglobules filled chloroplasts (Figure 8A,D). However, in the shade environment, chloroplasts were all arranged surrounding the plasma membrane and transformed from elliptically shaped to round (Figure 8B,C), and plastoglobules decreased or even disappeared completely (Figure 8D–F). The grana and thylakoids were abundant and unbroken under 10% light intensity and were injured to varying degrees under 40% light and full light intensities (Figure 8G–I). Statistical analyses demonstrated that shade significantly increased CA, CL, and CW (Table 5). Under 10% light intensity, CA, CL, and CW increased by 152.99%, 50.00%, and 95.14% compared to those under 40% light intensity, respectively.

### 2.6. RDPI Analysis

**Figure 9:**
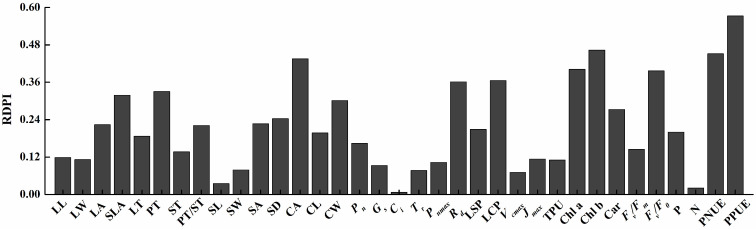
The relative distance phenotypic plasticity index of *P. arguta* seedlings under varied light intensities. LL, LW, LA, SLA, LT, PT, ST, PT/ST, SL, SW, SA, SD, CA, CL, CW, *P_n_*, *G_s_*, *C_i_*, *P_max_*, *R_d_*, LSP, LCP, *V_cmax_*, *J_max_*, TPU, Chl a, Chl b, Car, *F_v_*/*F_m_*, *F_v_*/*F_o_*, P, N, PNUE, PPUE.

The RDPI ranged from 0.007 to 0.573, with a mean of 0.22. The RDPI calculations revealed that the leaf characteristics of *P. arguta* plants were significantly altered by the light conditions (Figure 9). In general, the plasticity of photosynthetic pigments, PPUE, and PNUE of plants was higher than that of leaf and stomatal morphology and photosynthesis parameters. Regarding leaf, stomatal, and chloroplast morphology plasticity, the highest plasticity index was registered for CA, followed by PT, SLA, and CW. For photosynthesis parameters plasticity, the highest plasticity index was registered for LCP, followed by *R_d_* and LSP. In photosynthetic pigments and Chl fluorescence parameters plasticity, the highest plasticity index was registered for Chl b, followed by Chl a, *F_v_*/*F_0_*, and Car. In terms of nutrient elements, PNUE, and PPUE plasticity, the highest plasticity index was registered for PPUE and PNUE. Among all parameters, the PPUE was the most plastic trait, followed by Chl b, PNUE, CA, Chl a, and *F_v_*/*F_0_*, while *C_i_*, N, SL, *V_cmax_*, and *T_r_* were the least adaptable ones.

### 2.7. Correlation Analysis

**Figure 10:**
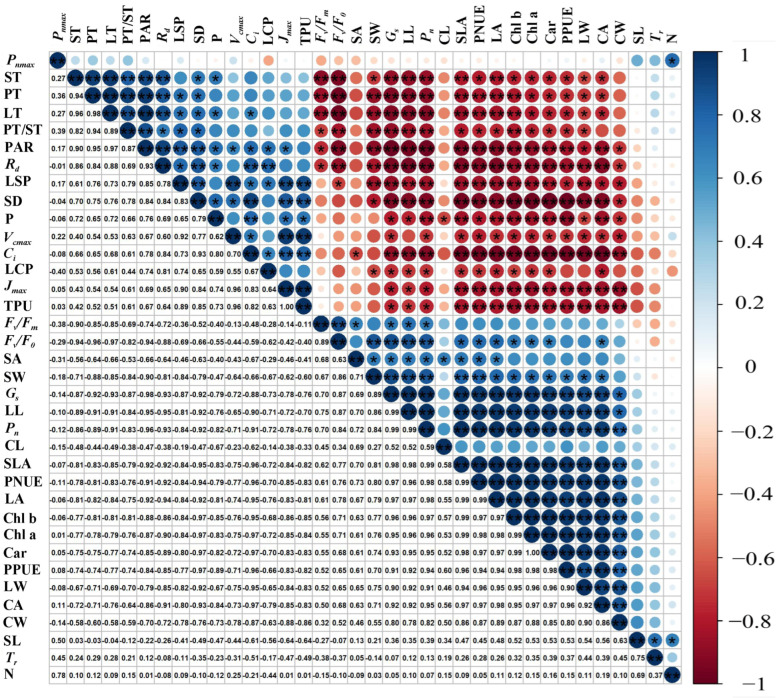
Pearson’s correlation coefficients across all indexes of *P. arguta* leaves under varied light intensities. * and ** indicate statistical significance at the 0.05 and 0.01 levels, respectively.

PAR was significantly correlated with the physiological morphological parameters of *P. arguta*, except TPU, SA, CL, SL, *T_r_*, N, and *P_nmax_* (Figure 10). *P_n_* was significantly positively correlated with CA, CW, LW, PPUE, Car, Chl a, Chl b, LA, PNUE, SLA, LL, *G_s_*, SW, SA, *F_v_*/*F_0_*, and *F_v_*/*F_m_*, and significantly negatively correlated with TPU, *J_max_*, LCP, *C_i_*, *V_cmax_*, P, SD, LSP, *R_d_*, PT/ST, LT, PT, and ST. However, *P_nmax_* was only positively correlated with N. Leaf shape parameters such as LL, LA, LW, and SLA were significantly positively correlated with Chl, Chl fluorescence, PPUE, and PNUE and negatively correlated with light response curve parameters, carbon dioxide curve parameters, and blade semi-thin slice parameters.

## 3. Discussion

### 3.1. Physiological Features of P. arguta Leaves in Response to Varied Light Intensities

Light is the foundation for plant photosynthesis, affecting assimilation ability, activation of key enzymes, the opening of stomata, and the development of photosynthetic apparatus [6,27]. In this study, the *P_n_* and *G_s_* of *P. arguta* leaves decreased significantly under full light and 40% light treatments, and the *C_i_* increased significantly, which was likely associated with non-stomatal factors rather than stomatal factors [27]. Similar results were identified in the endangered plants *Emmenopterys henryi* and *Ulmus elongata* [28,29]. This indicates that high light damages the photosynthetic organs of *P. arguta* seedlings, resulting in a decrease in the activities of related enzymes involved in photosynthesis, thereby reducing the photosynthetic capacity of mesophyll cells. Additionally, the photosynthetic capacity is also impacted by the utilization capacity of light and CO_2_ substrate concentration. LSP, LCP, and *R_d_* can reflect the light utilization of plants, while *V_cmax_*, *J_max_*, and TPU can reflect the CO_2_ use of plants [30,31]. It has been reported that plants with lower LCP, LSP, and *R_d_* values are considered an adaptation strategy to grow more efficiently under low light conditions [6]. In this study, we identified a significant decrease in LCP, LSP, and *R_d_* of *P. arguta* seedlings under 10% light intensity, while the *P_nmax_* had no significant difference. These findings suggest that *P. arguta* seedlings can more effectively improve the utilization capacity of low light, reduce the loss of photosynthetic products, and maintain the relative balance of carbon metabolism [32]. The endangered plant *C. nitidissima* also adapts to low light by reducing LSP, LCP, and *R_d_*, to maintain a good *P_n_* [8]. Generally, carbon assimilation is linked to Rubisco activity and ribulose-1,5-bisphosphate (RuBP) regeneration rate. The rate of *V_cmax_* in leaves is influenced by the activity and quantity of the Rubisco enzyme with fixed CO_2_. *J_max_* represents the regeneration capacity of RuBP and the electron transfer rate for RuBP regeneration [33]. Our findings indicated that the *J_max_* subjected to 10% light intensity was lower than those of other treatments, while the *V_cmax_* had no significant difference, suggesting that the *P. arguta* seedlings could maintain a relatively stable RuBP carboxylation ability and CO_2_ utilization capacity under low light intensity. This is consistent with the habitat conditions of the natural distribution of *P. arguta* population in the wild. The investigation of the *P. arguta* community structure showed that the plants generally lived in evergreen deciduous broad-leaved forests, distributed in patches in the forests, and the shade environment was obvious [34].

Measurement of photosynthetic pigments and Chl fluorescence parameters can indirectly reflect the photosynthetic capacity [35]. In this study, we identified a significant increase in the Chl contents of *P. arguta* under the 10% light intensity. Similar findings were observed in the endangered plant *Mahonia bodinieri* [36]. The elevation in pigment contents was conducive to the maintenance of high photosynthesis, predominantly by increasing the capture of diffuse light [35,37]. The Chl contents were the lowest under full light intensity compared to under shaded conditions, potentially caused by excessive radiation [38,39]. *F_v_*/*F_m_* is associated with environmental stress in plants. When *F_v_*/*F_m_* is below 0.80, it indicates that plants are in a state of environmental stress [40], simultaneously, the PSII reaction center is deactivated or damaged, causing a decrease in the *F_v_*/*F_0_*. Only the *F_v_*/*F_0_* between 4 and 6 can maintain the function of the photosystem II reaction center [41,42]. In the present study, severe shading (10% light intensity) maintained the *F_v_*/*F_0_* and *F_v_*/*F_m_* at normal levels, while full light significantly lowered them, indicating that photoinhibition occurred in *P. arguta* seedlings under full light, causing the PSII reaction center to be inactivated or damaged. In addition, the correlation of the *P_n_* with *F_v_*/*F_0_* and *F_v_*/*F_m_* was significant under the three light-intensity treatments. It is possible that *P. arguta* seedlings being under low light intensity contributes to improving the utilization rate of light energy.

Nitrogen (N) is a key component of photosynthetic protein (Rubisco), and leaf photosynthetic capacity is usually highly positively correlated with N content [43]. P is a pivotal component in protein synthesis due to its presence in ribosomal RNA [44]. Under the shade, most plants tend to synthesize more photosynthetic proteins to improve photosynthesis and increase tolerance, thus resulting in increased N and P in leaves [45]. In our study, no significant change in N under varied light exposure was observed, while P was significantly lowered under 10% light intensity. However, the *P_n_* of *P. arguta* seedlings peaked under 10% light intensity compared to other treatments, indicating that increasing P did not cause the promotion of ATP and NADPH generation, triose-phosphate exchange levels, and the renewal of Rubisco [46]. Additionally, leaf N and P also directly impact PNUE and PPUE, respectively [47]. PNUE and PPUE are considered to be important functional traits reflecting the physiological characteristics of leaves. Plants with greater PNUE tend to provide the photosynthetic system with more N and display marked growth rates [48]. In this study, the PNUE and PPUE of *P. arguta* seedlings peaked under 10% light intensity compared to other treatments, indicating that *P. arguta* seedlings maintained a higher photosynthetic rate by increasing the PNUE and PPUE. It is possible that a higher proportion of N and P was allocated in the photosynthetic system under low-light conditions. Similar results were observed in the endangered plant *U. elongate*, which modulates PNUE to promote the photosynthetic rate [29]. It can be seen that the shade tolerance mechanism of *P. arguta* seedlings is to increase PPUE and PNUE rather than the N and P contents.

### 3.2. Leaf Structure and Chloroplast Ultrastructure of P. arguta in Response to Varied Light Intensities

Leaves are the main photosynthetic organs with strong morphological plasticity [49]. Shade-tolerant plants can enhance their photosynthesis by increasing their LA and SLA under shade, so as to strike a balance between plant input and income as far as possible [8]. Here, our findings showed that the influence of light intensity on leaf traits was significant for *P. arguta*. The LL, LW, LA, and SLA were highest under 10% light intensity compared to other treatments. This was aligned with previous studies that showed leaf morphology increased under insufficient light intensity in *Phoebe bournei* and *Tetrastigma hemsleyanum* plants [42,50]. This adjustment enabled the plants to enhance the capture of light energy in a weak light environment. Stomata are important channels for gas exchange between plants and the external environment, including H_2_O and CO_2_. Their distribution density and size directly determine the transpiration and photosynthetic efficiency of plants [51]. The stomatal characteristics, encompassing SD, SA, SL, and SW, have changed adaptively in varied light environments. Generally, with decreased light intensity, the SD and SA decreased significantly [12,52]. In our study, the SD of *P. arguta* leaves reduced significantly, while the SA increased significantly under 10% light intensity. A marked positive correlation was observed between *P_n_* and stomatal properties (SA, SW). The findings of our study are inconsistent with previous findings, which demonstrated a decrease in the level of stomatal size in the endangered plants *H. miconioides* and *Sinopodophyllum hexandrum* leaves under low light intensity [6,12]. SA is associated with the efficiency of CO_2_ uptake and water loss reduction, leaf stomatal conductance, and photosynthetic rate [53,54]. Such changes in the SA of *P. arguta* seedlings may be caused by phenotypic changes and the maximization of carbon gain [55]. Hence, low light conditions encouraged the stomatal size development of *P. arguta* to maintain a high photosynthetic capacity.

The leaf anatomy structure, such as the thickness of PT and ST, the number of cell layers, and the morphology of palisade cells, affect carbon dioxide transport and ultimately affect photosynthesis [56]. Generally, thick leaves are suited to plant photosynthesis because thick leaves increase epidermal structure, PT, and ST, leading to the presence of more chloroplasts [57]. This study found that the LT, PT, ST, and PT/ST of the leaves in *P. arguta* seedlings were reduced significantly along with the increase in shade, consistent with the response of *H. miconioides* and *Corylus avellana* seedlings to varied light intensities [6,58]. Previous studies have proposed that thinner leaves brought mesophyll cells nearer to the epidermis, reducing the diffusion distance for CO_2_ from the outside to chloroplasts, and lowering the distance necessary for light to penetrate leaves [59]. The reduction in PT and ST thickness lowered the area of mesophyll, while *G_s_* and *P_n_* in leaves were increased significantly in 10% light condition, showing that CO_2_ concentration, rather than the photosynthetic reaction area, was the most significant factor under shaded conditions. In addition, it has also been suggested that thinner leaves may improve nutrient absorption rates [60], which is consistent with our findings of increased PPUE and PNUE under 10% light condition.

The thylakoids and grana in chloroplasts are the main sites of photosynthesis, and their intact structure and function of chloroplasts can ensure the efficient photosynthesis rate of plants [9]. In this study, *P. arguta* seedlings grown under 10% light condition contained more thylakoids, with better-developed grana than those of other treatments. This is generally consistent with the chloroplast structure found in *Torreya grandis* seedling leaves, which indicated that 75% and 90% shading treatments were beneficial for chloroplast development [5]. The more complete thylakoid and grana in leaves could assist in increasing the contents of PSII complexes and light-harvesting pigments, capture more light energy, and improve the absorption and conversion of light energy, which might be an important shading adaptation mechanism for *P. arguta* seedlings. The maintenance of stable thylakoid membranes under 10% light condition was also associated with high Car content [61]. In contrast, the destruction of thylakoid accumulation, the decomposition of the thylakoid membrane, and the enlargement of plastoglobules were observed under full light treatment, consistent with the decrease in photosynthetic rate. Plastoglobules in the chloroplast function in thylakoid formation, and their size and number can be used as indicators of chloroplast senescence [62,63]. Prior research has shown that the increase and formation of reactive oxygen species triggered by excessive light destroy the cell membrane structure in the chloroplasts of endangered plants, such as *Clematis tientaiensis* [64]. In addition, the micrographs of chloroplasts ultrastructure also indicated that starch size following exposure to the 10% light condition was larger than other treatments. This indicates the high photosynthetic capacity of their leaves. The elevation in starch may be closely linked to the maintenance of high concentrations of sugars close to chloroplast thylakoids and the regulation of chloroplast osmotic pressure [65,66].

### 3.3. RDPI and Correlation Analysis of P. arguta Leaves in Response to Varied Light Intensities

The adaptability of plant leaves results in the reaction of species to light treatments, and high phenotypic plasticity elevates the plant’s resistance to varied light environments [67]. In our study, the high plasticity of the photosynthetic, physiological, and phenotypic characteristics confirmed the shade tolerance of *P. arguta*. Nevertheless, the adaptability of the characteristics varied, and the primary trends exhibited higher physiological plasticity compared to morphological and anatomical features. This was also found in *Carpotroche brasiliensis* [68]. The five indices with the highest RDPI (PPUE, Chl b, PNUE, Chl a, and *F_v_*/*F_0_*) were physiological indices, and all were significantly positively correlated with *P_n_* (r > 0.90). This indicates that the high *P_n_* maintained by *P. arguta* seedlings under 10% light intensity is achieved by modulating these indices. Among the structural parameters, SLA is the most plastic and is also significantly positively correlated with *P_n_* (r = 0.99). Regulating SLA is a method of maintaining high *P_n_* in *P. arguta* seedlings. Generally, photosynthetic, physiological, and phenotypic characteristics of *P. arguta* restrict and influence each other under varied shading environments.

## 4. Materials and Methods

### 4.1. Plant Material and Treatments

**Figure 11:**
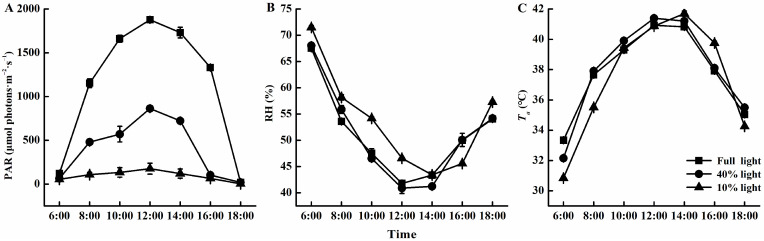
Daytime changes of photosynthetically active radiation (PAR), relative humidity (RH), and air temperature (*T_a_*) under varied light intensities. (**A**) PAR, (**B**) RH, and (**C**) *T_a_*.

Experiments were performed in a greenhouse at Taizhou University, Zhejiang Province, China (longitude: 127°17′ E; latitude: 28°87′ N). We employed individual three-year-old *P. arguta* seedlings in this study. The seedlings were grown in round plastic pots with a top diameter of 23.50 cm, a bottom diameter of 20.80 cm, and a height of 26.30 cm. The substrate used was moist vegetative soil (peat soil: paddy soil: river sand = 6:6:1, *v*/*v*/*v*). The light control experiment was performed under a sunshade canopy built with black sunshade nets, under three treatments: no sunshade coverage (light intensity of 100% or full light), a layer of sunshade net coverage (light intensity of approximately 40% of full light), and two layers of sunshade net coverage (light intensity of approximately 10% of full light). After 3 months of shading experiments, all parameters were sampled and measured within one week. In addition, we assessed the daytime alterations in photosynthetically active radiation (PAR), air temperature (*T_a_*), and relative humidity (RH) across three varied light exposures during sunny days for 3 consecutive days (Figure 11).

### 4.2. Measurement of Photosynthetic gas Exchange Parameters

In July 2019, the photosynthetic features of leaves were examined for in situ photosynthetic attributes utilizing a portable LI-6400 XT photosynthetic system (Li-Cor, Lincoln, NE, USA) on a sunny day from 09:00 to 11:30. The net photosynthetic rate (*P_n_*, μmol·m^-2^·s^-1^), stomatal conductance (*G_s_*, mmol·m^−2^·s^−1^), transpiration level (*T_r_*, mmol·m^−2^·s^−1^), and intercellular CO_2_ concentration (*C_i_*, μmol·mol^−1^) were identified under a PAR of 1000 μmol·m^−2^·s^−1^, which was maintained using an LED red/blue light source (6400-02B, Lincoln, NE, USA) at 25 °C with a maintained humidity of 70% RH and 400 ± 5 μmol·mol^−1^ CO_2_ concentration. Three plants derived from each treatment group were randomly chosen for repeated examinations of three leaves from each plant.

Photosynthetic and CO_2_ response curves were constructed under the same conditions as the photosynthetic characteristics. For light response curves, healthy, fully expanded leaves were investigated between 09:30 and 11:00 using the Li-6400XT. Prior to the determination, an LED red/blue light source was used for light induction to stabilize at a PAR of 2000 μmol·m^−2^·s^−1^ for 15–30 min. The PAR gradients were established at 2000, 1500, 1200, 1000, 800, 600, 400, 200, 150, 100, 50, 20, and 0 μmol·m^−2^·s^−1^. For CO_2_ response curves, the saturated light intensity of leaves was maintained at 1000 μmol·m^−2^·s^−1^, and CO_2_ concentration gradients were established at 1500, 1200, 1000, 800, 600, 400, 200, 150, 120, 100, 80, and 50 μmol·mol^−1^, and the maximum incubation time for each CO_2_ gradient was 300 s.

The light response curves were fitted using a modified rectangular hyperbola model [69,70]. According to the model, the corresponding predicted *P_n_* values and PAR-*P_n_* curves were acquired using nonlinear regression fitting using Origin 8.5. Photosynthetic parameters encompassing maximum net photosynthetic rate (*P_nmax_*, μmol·m^−2^·s^−1^), light saturation point (LSP, μmol·m^−2^·s^−1^), light compensation point (LCP, μmol·m^−2^·s^−1^), and dark respiration rate (*R_d_*, μmol·m^−2^·s^−1^) were calculated. The CO_2_ response curves were fitted using a non-rectangular hyperbolic model [71]. According to the model, the maximum Rubisco carboxylation rate (*V_cmax_*, μmol·m^−2^·s^−1^), maximum electron transport rate (*J_max_*, μmol·m^−2^·s^−1^), and triose-phosphate utilization (TPU, μmol·m^−2^·s^−1^) were determined.

### 4.3. Measurement of Chlorophyll Fluorescence Characteristics and Photosynthetic Pigments 

The chlorophyll (Chl) fluorescence was measured between 09:00 and 11:30 using a portable Chl fluorescence analyzer (MINI-PAM, Walz, Effeltrich, Germany), investigating the third healthy, fully developed leaf of *P. arguta* seedlings. Leaves were exposed to darkness 30 min prior to investigation. After identifying the initial fluorescence intensity (*F_o_*), maximum fluorescence intensity (*F_m_*), and variable fluorescence (*F_v_*), the activity of PSII reaction centers (*F_v_*/*F_o_*), and maximum photochemical efficiency of the PS II (*F_v_*/*F_m_*) were computed as described by Maxwell and Johnson [72].

For Chl level assessment, a 0.2 g sample of the third leaf blade was weighed, crushed, and incubated in 5 mL of 80% acetone (*v*/*v*) in the dark at room temperature. The optical density (OD) of the supernatant was examined using a UV-vis spectrophotometer (T6 New Century, Beijing, China) at 470 nm (OD_470_), 663 nm (OD_663_), and 645 nm (OD_645_). According to Lichtenthaler and Wellburn’s [73] instructions, the contents of Chl a, Chl b, and Car were determined using the following formulas:

(1) $$Chl\;a=(12.72\times OD_{663}-2.59\times OD_{645})\times V/1000\times W$$

(2) $$Chl\;b=(22.88\times OD_{645}-4.67\times OD_{663})\times V/1000\times W$$

(3) $$Car=[4.7\times OD_{470}-0.27\times \left(Chla-Chlb\right)]\times V/1000\times W$$

where V represents the total volume of acetone extract (mL), and W denotes the fresh weight of the sample (g).

### 4.4. Determination of Leaf Traits and Nutrient Elements

In July 2019, we obtained 15 leaves from each treatment and then measured the leaf area (LA, cm^2^), leaf length (LL, cm), and leaf width (LW, cm) using WinFOLIA (Regent Instruments, Inc., Québec City, QC, Canada). After, these leaves were placed in an oven at 80 °C until they reached a constant weight. Subsequently, we weighed these leaves and computed the SLA (cm^2^·g^−1^):

SLA = LA/leaf biomass(4)

These dried leaves were pulverized into a powder and passed through a screen with a 1 mm mesh. A sample of 0.25 g of leaf powder was digested and diluted in H_2_SO_4_-H_2_O_2_ solution, and the unit mass of leaf nitrogen (N, mg·g^−1^) and phosphorus (P, mg·g^−1^) were measured using an AA3 Flow analyzer (AA3, Seal Analytical, Norderstedt, Germany). The photosynthetic nitrogen use efficiency (PNUE, μmol·g^−1^·s^−1^) and photosynthetic phosphorus use efficiency (PPUE, μmol·g^−1^·s^−1^) were determined as follows [74]:

PNUE = *P_n_*/[(N/SLA) × 10] (5)

PPUE = *P_n_*/[(P/SLA) × 10] (6)

### 4.5. Characterization of Leaf Anatomy, Stomata, and Chloroplast Ultrastructure

For leaf anatomy observation, leaf segments (5 mm × 5 mm) lacking veins were fixed with FAA solution (38% formaldehyde, glacial acetic acid, 70% alcohol, 5:5:90, *v*/*v*/*v*) (over 12 h) and dehydrated with an alcohol gradient (30 min each in 50%, 60%, 70%, 85%, 95% and 1 h in 100%). The samples were soaked in propylene oxide and Spurr’s epoxy resin, which was allowed to polymerize at 65 °C for 48 h. The samples were sectioned using a Leica EM UC7 ultramicrotome (Leica, Wetzlar, Hessian, Germany) and stained using 0.05% toluidine blue solution for 5 minutes. The sections were observed and photographed using a Leica DM2500 microscope (Leica, Wetzlar, Hessian, Germany). The embedding and observation processes were performed using the previous method with some modifications [75]. Subsequently, measurements of leaf thickness (LT, μm), palisade tissue (PT, μm), and spongy tissue (ST, μm) were performed using ImageJ 1.8.0 (Bethesda, Maryland, USA), and the ratio of palisade tissue to spongy tissue (PT/ST) was determined.

For leaf stomatal observation, fresh leaves (5 mm × 5 mm) were obtained, fixed, and dehydrated, mirroring the steps used to characterize the leaf anatomy. Subsequently, the sample was treated with critical point drying (EM CPD300, Leica, Wetzlar, Hessian, Germany), and the stomatal status of leaves was identified using an S-4800 microscope (Hitachi, Tokyo, Japan). The stomatal length (SL, μm), stomatal width (SW, μm), and stomatal aperture (SA, μm) were characterized using ImageJ 1.8.0, and the number of stomata per square millimeters was identified as stomatal density (SD, n·mm^−2^).

For chloroplast ultrastructural observation, the functional leaves (5 mm × 5 mm) were soaked in a 2.5% glutaraldehyde fixative solution, evacuated using a vacuum pump, and stored at 4 °C. The samples were rinsed with phosphate buffer, immersed in 1.0% osmium acid, and dehydrated utilizing an ascending alcohol series (50–100%). The samples were immersed and polymerized. Ultrathin sections were obtained using a Leica EM UC7 ultramicrotome and double stained using uranyl acetate–lead citrate before imaging using a transmission electron microscope (JEOL JEM-1300, Tokyo, Japan). The embedding and observation processes were performed as the previous method with some modifications [75]. Finally, the chloroplast area (CA, μm^2^), chloroplast length (CL, μm), and chloroplast width (CW, μm) were characterized using ImageJ 1.8.0.

### 4.6. Determination of Relative Distance Phenotypic Plasticity Index (RDPI)

To identify traits responsible for plastic responses of *P. arguta* to varied light intensities, the RDPI proposed by Valladares et al. [76] was employed. Here, the RDPI ranges from 0 to 1, and its value closer to 1 is considered an indicator of high plasticity.

(7) $$RDPI=\sum [d_{ij\to i^{\prime }j^{\prime }}/(x_{i^{\prime }j^{\prime }}+x_{ij})]/n$$

where *j* and *j′* represent the individuals, *i* and *i′* denote the environments, $d_{ij\to i^{\prime }j^{\prime }}/(x_{i^{\prime }j^{\prime }}+x_{ij})$ represents the relative distance for the pairs of individuals exposed to varied environments, and n indicates the total number of distances.

### 4.7. Data Analysis

In order to investigate the suitable light intensities for plant growth and photosynthetic activity, all physiological and structural data were analyzed via one-way ANOVA using SPSS 18.0 software (SPSS Inc., Chicago, Illinois, USA), and data distribution normality was determined prior to analysis. Multiple comparisons were performed using the LSD method for homogeneity of variance and Dunnett’s T3 method for heterogeneity of variance. The data presented are means ± standard. All figures were plotted using Origin 8.5 software (Origin Lab, Northampton, MA, USA). Correlation analysis was performed to explore the correlation between the measured data using R version 3.2.3 (R Development Core Team 2016), and the correlation values were presented in a correlation heatmap.

## 5. Conclusions

According to the findings of leaf phenotype, photosynthesis, physiological characteristics, and chloroplast structure, we can confirm that *P. arguta* is a shade-adapted plant with limited adaptability to high-light environments. The *P. arguta* seedlings had no obvious photoinhibition under 10% light intensity, while under 40% light and full light intensities, varying levels of photoinhibition were identified and were more pronounced under full light exposure. This was mainly displayed in higher LA, SLA, *T_r_*, *G_s_*, Chl, *F_v_*/*F_m_*, *F_v_*/*F_0_*, PNUE, PPUE, SL, SA, and CA, but lower LCP, TPU, *R_d_*, P, and LT under 10% light intensity. Simultaneously, the number of chloroplast grana and thylakoids was increased under 10% light intensity. The *P. arguta* seedlings maintained higher *P*_n_ with higher plasticity for physiological variables than for morphological and anatomical variables, especially PPUE, Chl b, PNUE, Chl a, and *F_v_*/*F_o_*. Therefore, we propose that the suitable growth condition of *P. arguta* was 10% light intensity, and seedlings maintained optimal photosynthesis by adjusting phenotypic characteristics, photosynthetic physiological characteristics, and chloroplast structure, increasing their photosynthetic pigments and leaf area, and forming complete chloroplast structures. The future work will be focused on comparative investigation of the adaptation mechanisms of different light-demanding plants (such as shade plants and sun plants) in their specific environment.

## Figures and Tables

**Table 1:** Leaf phenotypic parameters of *P. arguta* under varied light intensities.

Treatments	Leaf Length (LL)/cm	Leaf Width (LW)/cm	Leaf Area (LA)/cm^2^	Specific Leaf Area (SLA)/cm^2^·g^−1^
Full light	11.04 ± 0.75 c	4.04 ± 0.14 c	25.83 ± 2.63 c	88.68 ± 1.73 c
40% light	13.31 ± 0.44 b	4.54 ± 0.14 b	35.12 ± 1.82 b	134.80 ± 3.35 b
10% light	16.48 ± 0.22 a	6.01 ± 0.18 a	54.86 ± 2.42 a	243.21 ± 4.81 a

Data are displayed as the mean ± standard deviation. Different lowercase letters in the same column denote significant differences among different treatments at the 0.05 level.

**Table 2:** The alterations in photosynthetic parameters of *P. arguta* under varied light intensities.

Parameters	Full Light	40% Light	10% Light
*P_nmax_* (µmol·m^−2^·s^−1^)	7.73 ± 1.62 a	6.40 ± 1.17 a	7.27 ± 0.70 a
LCP (µmol·m^−2^·s^−1^)	32.46 ± 16.12 a	16.56 ± 0.58 ab	6.38 ± 2.16 b
LSP (µmol·m^−2^·s^−1^)	841.33 ± 178.45 a	666.67 ± 112.88 ab	445.33 ± 35.85 b
*R_d_* (µmol·m^−2^·s^−1^)	1.65 ± 0.34 a	1.14 ± 0.07 b	0.51 ± 0.15 c
*V_cmax_* (µmol·m^−2^·s^−1^)	30.35 ± 1.89 a	29.45 ± 1.48 a	25.32 ± 0.14 a
*J_max_* (µmol·m^−2^·s^−1^)	28.75 ± 2.28 a	28.66 ± 0.94 a	21.41 ± 0.17 b
TPU (µmol·m^−2^·s^−1^)	6.93 ± 0.55 a	6.98 ± 0.25 a	5.24 ± 0.05 b

Data are displayed as the mean ± standard deviation. Different lowercase letters in the same column indicate significant differences among treatments at the 0.05 level.

**Table 3:** Variations in the mean stomatal structure of *P. arguta* leaves under different light intensities.

Treatments	Stomatal Length (SL)/μm	Stomatal Width (SW)/μm	Stomatal Aperture (SA)/μm^2^	Stomatal Density (SD)/n·mm^−2^
Full light	21.07 ± 0.59 ab	11.75 ± 0.26 b	1.68 ± 0.30 b	528.33 ± 15.74 a
40% light	19.83 ± 0.46 b	13.46 ± 0.37 a	2.28 ± 0.17 b	512.50 ± 34.27 a
10% light	21.75 ± 0.41 a	14.68 ± 0.42 a	3.11 ± 0.22 a	262.50 ± 37.35 b

Data are displayed as the mean ± standard deviation. Different lowercase letters in the same column denote significant differences among different treatments at the 0.05 level.

**Table 4:** The alterations in anatomical characteristics of *P. arguta* grown under varied light intensities.

Treatments	Leaf Thickness (LT)/μm	Palisade Tissue (PT)/μm	Spongy Tissue (ST)/μm	Palisade Tissue /Spongy Tissue (PT/ST)
Full light	155.63 ± 1.48 a	54.16 ± 1.38 a	85.47 ± 2.10 a	0.64 ± 0.03 a
40% light	102.13 ± 0.95 b	26.09 ± 1.29 b	63.54 ± 1.51 b	0.42 ± 0.03 b
10% light	87.89 ± 1.08 c	18.92 ± 0.76 c	56.97 ± 1.38 c	0.34 ± 0.02 c

Data are displayed as the mean ± standard deviation. Different lowercase letters in the same column denote significant differences among different treatments at the 0.05 level.

**Table 5:** The alterations in chloroplast characteristics of *P. arguta* grown under varied light intensities.

Treatments	Chloroplast Area (CA)/μm^2^	Chloroplast Length (CL)/μm	Chloroplast Width (CW)/μm
Full light	8.00 ± 1.18 b	4.53 ± 0.47 b	2.27 ± 0.19 b
40% light	12.34 ± 1.72 b	4.66 ± 0.29 b	2.88 ± 0.21 b
10% light	31.22 ± 2.11 a	6.99 ± 0.25 a	5.62 ± 0.31 a

Data are displayed as the mean ± standard deviation. Different lowercase letters in the same column denote significant differences among different treatments at the 0.05 level.

## Data Availability

Data recorded in the current study are available in all tables and figures of the manuscript.

## References

[1] Valladares F., Wright S.J., Lasso E., Kitajima K., Pearcy R.W. (2000). Plastic Phenotypic Response to Light of 16 Congeneric Shrubs from a Panamanian Rainforest. Ecology.

[2] Zhao D., Hao Z., Tao J. (2012). Effects of shade on plant growth and flower quality in the herbaceous peony (*Paeonia lactiflora* Pall.). Plant Physiol. Bioch..

[3] Lu D.Y., Liu B., Ren M.J., Wu C., Ma J.J., Shen Y.M. (2021). Light Deficiency Inhibits Growth by Affecting Photosynthesis Efficiency as well as JA and Ethylene Signaling in Endangered Plant *Magnolia sinostellata*. Plants.

[4] Sharma N., Nagar S., Thakur M., Suriyakumar P., Kataria S., Shanker A.K., Landi M., Anand A. (2023). Photosystems under high light stress: Throwing light on mechanism and adaptation. Photosynthetica.

[5] Tang H., Hu Y.Y., Yu W.W., Song L.L., Wu J.S. (2015). Growth, photosynthetic and physiological responses of *Torreya grandis* seedlings to varied light environments. Trees.

[6] Zhang Y.F., Chen C., Jin Z.X., Yang Z.N., Li Y.L. (2022). Leaf anatomy, photosynthesis, and chloroplast ultrastructure of *Heptacodium miconioides* seedlings reveal adaptation to light environment. Environ. Exp. Bot..

[7] Lu X.H., Xu N., Chen Y., Li Y., Gan X.H. (2021). Effects of light intensity and ground cover on seedling regeneration of *Tetracentron sinense* Oliv. J. Plant Growth Regul..

[8] Chai S.F., Tang J.M., Mallik A., Shi Y.C., Zou R., Li J.T., Wei X. (2018). Eco-physiological basis of shade adaptation of *Camellia nitidissima*, a rare and endangered forest understory plant of Southeast Asia. BMC Ecol..

[9] Ma X.H., Song L.L., Yu W.W., Hu Y.Y., Liu Y., Wu J.S., Ying Y.Q. (2015). Growth, physiological, and biochemical responses of *Camptotheca acuminata* seedlings to different light environments. Front. Plant Sci..

[10] Li D.L., Jin Y.Q., Cui M.F., Wang H., Jiang H., Zhu Y.Y. (2019). Photosynthetic Characteristics and Leaf Anatomical Structure of *Cercidiphyllum japonicum* Seedlings under Shading Condition. Acta Bot. Boreali-Occident. Sin..

[11] Lockhart B.R., Gardiner E.S., Stautz T., Leininger T.D. (2012). Development and plasticity of endangered shrub *Lindera melissifolia* (Lauraceae) seedlings under contrasting light regimes. Plant Species Biol..

[12] Guo Q.Q., Li H.E., Gao C., Yang R. (2019). Leaf traits and photosynthetic characteristics of endangered *Sinopodophyllum hexandrum* (Royle) Ying under different light regimes in Southeastern Tibet Plateau. Photosynthetica.

[13] Zhou Y., Huang L.H., Wei X.L., Zhou H.Y., Xun C. (2017). Physiological, morphological, and anatomical changes in *Rhododendron agastum* in response to shading. Plant Growth Regul..

[14] Liu Q.Q., Huang Z.J., Wang Z.N., Chen Y.F., Wen Z.M., Liu B., Tigabu M. (2020). Responses of leaf morphology, NSCs contents and C:N:P stoichiometry of *Cunninghamia lanceolata* and *Schima superba* to shading. BMC Plant Biol..

[15] Zhang Y.Y., Yu T., Ma W.B., Tian C., Sha Z.P., Li J.Q. (2019). Morphological and physiological response of *Acer catalpifolium* Rehd. seedlings to water and light stresses. Glob. Ecol. Conserv..

[16] Shao Q.Q., Wang H.Z., Guo H.P., Zhou A., Huang Y.Q., Sun Y.L., Li M.Y. (2014). Effects of shade treatments on photosynthetic characteristics, chloroplast ultrastructure, and physiology of *Anoectochilus roxburghii*. PLoS ONE.

[17] Pfeiffer S., Krupinska K. (2005). New insights in thylakoid membrane organization. Plant Cell Physiol..

[18] Canham C.D., Kobe R.K., Latty E.F., Chazdon R.L. (1999). Interspecific and intraspecific variation in tree seedling survival: Effects of allocation to roots versus carbohydrate reserves. Oecologia.

[19] Wei C.Y., Wang Q.Q., Han H.Y., Gan X.H. (2023). Low soil moisture improved shading tolerance by regulating leaf functional traits in *Tetracentron sinense* Oliv. seedlings. Glob. Ecol. Conserv..

[20] Wei Z.F., Bruce B., Wu Z.Y., Raven P.H. (2001). Flora of China.

[21] Qi X.S., Yuan N., Comes P.H., Sakaguchi S., Qiu Y.X. (2014). A strong ‘filter’ effect of the East China Sea land bridge for East Asia’s temperate plant species: Inferences from molecular phylogeography and ecological niche modelling of *Platycrater arguta* (Hydrangeaceae). BMC Evol. Biol..

[22] Wen J.X., Hu Q.D., Ma X.H., Ye Y.J., Chen Z.Y., Chen Q.X., Zheng J., Qian R.J. (2023). Advances in studies on the endangered plant *Platycrater arguta*. J. Zhejiang Agri. Sci..

[23] Fu L.G. (1991). China Plant Red Data Book: Rare and Endangered Plants.

[24] Ge L.P., Lu A.M., Gong C.R. (2007). Ontogeny of the fertile flower in *Platycrater arguta* (Hydrangeaceae). Int. J. Plant Sci..

[25] Ao C.Q. (2008). Pre-zygotic embryological characters of *Platycrater arguta*, a rare and endangered species endemic to East Asia. J. Plant Bio..

[26] Li Y.L., Wei C.Y., Chen X.Y., Sun Z.S. (2020). Characterization of the complete chloroplast genome sequence of the endangered species *Platycrater arguta* (Hydrangeaceae). Mitochondrial DNA B.

[27] Farquhar G.D., Sharkey T.D. (1982). Stomatal conductance and photosynthesis. Ann. Rev. Plant. Physiol..

[28] Li D.L., Jin Y.Q., Cui M.F., Huang L.X., Pei W.H. (2020). Effects of shading on physiological characteristics and ultrastructure of mesophyll cell of *Emmenopterys henryi* leaves. Bull. Bot. Res..

[29] Luo G.Y., Li J.M., Guo S.L., Li Y.L., Jin Z.X. (2022). Photosynthesis, nitrogen allocation, non-structural carbohydrate allocation, and C:N:P stoichiometry of *Ulmus elongata* seedlings exposed to different light intensities. Life.

[30] Jacob J., Greitner C., Drake B.G. (1995). Acclimation of photosynthesis in relation to Rubisco and non-structural carbohydrate contents and in situ carboxylase activity in *Scirpus olneyi* grown at elevated CO_2_ in the field. Plant Cell Environ..

[31] Calzavara A.K., Bianchini E., Pimenta J.A., Oliveira H.C., Stolf-Moreira R. (2019). Photosynthetic light-response curves of light-demanding and shade-tolerant seedlings of neotropical tree species. Photosynthetica.

[32] Meng F.Q., Cao R., Yang D.M., Niklas K.J., Sun S.C. (2013). Within-twig leaf distribution patterns differ among plant life-forms in a subtropical Chinese forest. Tree Physiol..

[33] Ichikawa K., Miyake C., Iwano M., Sekine M. (2008). Ribulose 1,5-bisphosphate carboxylase/oxygenase large subunit translation is regulated in a small subunit-independent manner in the expanded leaves of tobacco. Plant Cell Physiol..

[34] Zhang L.F., Qiu L.H. (2017). Flowering phenology, floral traits and breeding system of *Platycrater arguta*. Guihaia.

[35] Mao L.Z., Lu H.F., Wang Q., Cai M.M. (2007). Comparative photosynthesis characteristics of *Calycanthus chinensis* and *Chimonanthus praecox*. Photosynthetica.

[36] Kong D.X., Li Y.Q., Wang M.L., Bai M., Zou R., Tang H., Wu H. (2016). Effects of light intensity on leaf photosynthetic characteristics, chloroplast structure, and alkaloid content of *Mahonia bodinieri* (Gagnep.) Laferr. Acta Physiol. Plant..

[37] Flores-de-Santiago F., Kovacs J.M., Wang J.F., Flores-Verdugo F., Zhang C.H., González-Farías F. (2016). Examining the influence of seasonality, condition, and species composition on mangrove leaf pigment contents and laboratory based spectroscopy data. Remote Sens..

[38] Sato R., Ito H., Tanaka A. (2015). Chlorophyll b degradation by chlorophyll b reductase under high-light conditions. Photosynth. Res..

[39] Zhang H.H., Zhong H.X., Wang J.F., Sui X., Xu N. (2016). Adaptive changes in chlorophyll content and photosynthetic features to low light in *Physocarpus amurensis* Maxim and *Physocarpus opulifolius* “Diabolo”. Peer J..

[40] Duan R.Y., Huang M.Y., Kong X.Q., Wang Z.G., Fan W.Y. (2015). Ecophysiological responses to different forest patch type of two codominant tree seedlings. Ecol. Evol..

[41] Rohacek K. (2002). Chlorophyll fluorescence parameters: The definitions, photosynthetic meaning, and mutual relationships. Photosynthetica.

[42] Dai Y.J., Shen Z.G., Liu Y., Wang L.L., Hannaway D., Lu H.F. (2009). Effects of shade treatments on the photosynthetic capacity, chlorophyll fluorescence, and chlorophyll content of *Tetrastigma hemsleyanum* Diels et Gilg. Environ. Exp. Bot..

[43] Asif I., Niu J., Dong Q., Wang X.R., Gui H.P., Zhang H.H., Pang N.C., Zhang X.L., Song M.Z. (2022). Physiological characteristics of cotton subtending leaf are associated with yield in contrasting nitrogen efficient cotton genotypes. Front. Plant Sci..

[44] Li H.L., Crabbe M.J.C., Xu F.L., Wang W.L., Ma L.H., Niu R.L., Gao X., Li X.X., Zhang P., Ma X. (2017). Seasonal variations in carbon, nitrogen and phosphorus concentrations and C:N:P stoichiometry in different organs of a *Larix principis-rupprechtii* Mayr. plantation in the Qinling Mountains, China. PLoS ONE.

[45] Poorter L., Bongers F. (2006). Leaf traits are good predictors of plant performance across 53 rain forest species. Ecology.

[46] Santos M.G., Ribeiro R.V., Oliveira R.F., Pimentel C. (2004). Gas exchange and yield response to foliar phosphorus application in Phaseolus vulgaris L. under drought. Braz. J. Plant Physiol..

[47] Katahata S., Naramoto M., Kakubari Y., Mukai Y. (2007). Photosynthetic capacity and nitrogen partitioning in foliage of the evergreen shrub, *Daphniphyllum humile*, along a natural light gradient. Tree Physiol..

[48] Poorter H., Remkes C., Lambers H. (1990). Carbon and nitrogen economy of 24 wild species differing in relative growth rate. Plant Physiol..

[49] Uemura A., Ishida A., Nakano T., Terashima I., Tanabe H., Matsumoto Y. (2000). Acclimation of leaf characteristics of Fagus species to previous-year and current-year solar irradiances. Tree Physiol..

[50] Tang X.L., Liu G.Z., Jiang J., Lei C.J., Liu X.L. (2020). Effects of growth irradiance on photosynthesis and photorespiration of *Phoebe bournei* leaves. Funct. Plant Biol..

[51] Sack L., Buckley T.N. (2016). The developmental basis of stomatal density and flux. Plant Physiol..

[52] Pigliucci M., Kolodynska A. (2002). Phenotypic plasticity to light intensity in Arabidopsis thaliana: Invariance of reaction norms and phenotypic integration. Evol. Ecol..

[53] Büssis D., Groll V.U., Fisahn J., Altmann T. (2006). Stomatal aperture can compensate altered stomatal density in Arabidopsis thaliana at growth light conditions. Funct. Plant Biol..

[54] Drake P.L., Froend R.H., Franks P.J. (2012). Smaller, faster stomata: Scaling of stomatal size, rate of response, and stomatal conductance. J. Exp. Bot..

[55] Pilahome W., Bunnag S., Suwanagul A. (2017). Two-step salt stress acclimatization confers marked salt tolerance improvement in four rice genotypes differing in salt tolerance. Arab. J. Sci. Eng..

[56] Lu Z.F., Xie K.L., Pan Y.H., Ren T., Lu J.W., Wang M., Shen Q.R., Guo S.W. (2019). Potassium mediates coordination of leaf photosynthesis and hydraulic conductance by modifications of leaf anatomy. Plant Cell Environ..

[57] Fan Y.F., Chen J.X., Wang Z.L., Tan T.T., Li S.L., Li J.F., Wang B.B., Zhang J.W., Cheng Y.J., Wu X.L. (2019). Soybean (*Glycine max* L. Merr.) Seedlings response to shading: Leaf structure, photosynthesis and proteomic analysis. BMC Plant Biol..

[58] Catoni R., Granata M.U., Sartori F., Varone L., Gratani L. (2015). Corylus avellana responsiveness to light variations: Morphological, anatomical, and physiological leaf trait plasticity. Photosynthetica.

[59] Zhang C.H., Moutinho-Pereira J.M., Correia C., Coutinho J., Goncalves A., Guedes A., Gomes-Laranjo J. (2013). Foliar application of Sili-K (R) increases chestnut (*Castanea* spp.) growth and photosynthesis, simultaneously increasing susceptibility to water deficit. Plant Soil.

[60] Takeshi H., Masamitsu W. (2016). Chloroplast avoidance movement is not functional in plants grown under strong sunlight. Plant Cell Environ..

[61] Biswal B. (1995). Carotenoid catabolism during leaf senescence and its control by light. J. Photoch. Photobio. B.

[62] Roberto E., Steffen H., Andreas K., Peter J., Martin L., Jörg M., Jörg N., Jürgen S., Serena S. (2019). Plastoglobular protein 18 is involved in chloroplast function and thylakoid formation. J. Exp. Bot..

[63] Günthardt-Goerg M.S., Schläpfer R., Vollenweider P. (2023). Responses to airborne ozone and soilborne metal pollution in afforestation plants with different life forms. Plants.

[64] Ma X.H., Zhou Q., Hu Q.D., Zhang X.L., Zheng J., Qian R.J. (2023). Effects of different irradiance conditions on photosynthetic activity, photosystem II, rubisco enzyme activity, chloroplast ultrastructure, and chloroplast-related gene expression in *Clematis tientaiensis* leaves. Horticulturae.

[65] Wu Q.S., Srivastava A.K., Zou Y.N. (2013). AMF-induced tolerance to drought stress in citrus: A review. Sci. Hortic..

[66] Li Y.L., Wang X.Y., Chen X.Y., Lu J.Y., Jin Z.X., Li J.M. (2023). Functions of arbuscular mycorrhizal fungi in regulating endangered species *Heptacodium miconioides* growth and drought stress tolerance. Plant Cell Reports.

[67] Sultan S.E. (1995). Phenotypic plasticity and plant adaptation. Acta Bot. Neerl..

[68] Cerqueira A.F., Dalmolin Â.C., Anjos L.D., Ledo C.A.S., Silva D.C., Mielke M.S. (2018). Photosynthetic plasticity of young plants of *Carpotroche brasiliensis* (Raddi) A. Gray, Achariaceae. Trees.

[69] Marshall B., Biscoe P.V. (1980). A model for C3 leaves describing the dependence of net photosynthesis on irradiance. J. Exp. Bot..

[70] Ye Z.P. (2007). A new model for relationship between irradiance and the rate of photosynthesis in *Oryza sativa*. Photosynthetica.

[71] Long S.P., Bernacchi C.J. (2003). Gas exchange measurements, what can they tell us about the underlying limitations to photosynthesis? Procedures and sources of error. J. Exp. Bot..

[72] Maxwell K., Johnson G.N. (2000). Chlorophyll fluorescence: Apractical guide. J. Exp. Bot..

[73] Lichtenthaler H.K., Wellburn A.R. (1983). Determination of total carotenoids and chlorophylls a and b of leaf extracts in different solvents. Biochem. Soc. Trans..

[74] Field C., Mooney H.A., Givnish T.J. (1986). On the Economy and Form of Plant Function. The Photosynthesis-Nitrogen Relationship in Wild Plants.

[75] Zhang Z.B., Zhu J., Gao J.F., Wang C., Li H., Li H., Zhang H.Q., Zhang S., Wang D.M., Wang Q.X. (2007). Transcription factor AtMYB103 is required for anther development by regulating tapetum development, callose dissolution and exine formation in *Arabidopsis*. Plant J..

[76] Valladares F., Sanchez-Gomez D., Zavala M.A. (2006). Quantitative estimation of phenotypic plasticity: Bridging the gap between the evolutionary concept and its ecological applications. J. Ecol..

